# Expanding the genotypic and phenotypic spectrum of Egyptian children with maple syrup urine disease

**DOI:** 10.1038/s41598-024-78105-y

**Published:** 2024-11-18

**Authors:** Zeinab S. Abdelkhalek, Shadia M. Hussein, Iman G. Mahmoud, Areef Ramadan, Mona A. Kamel, Marian Y. Girgis, Mohamed A. Elmonem

**Affiliations:** 1 Clinical and Chemical Pathology Department, Faculty of Medicine, Cairo University, Cairo University Children’s Hospital, Monira, 11628 Cairo Egypt; 2 Central laboratories, Cairo University Hospitals, Cairo University, Cairo, Egypt; 3 Pediatric Neurology Department, Metabolic Division, Faculty of Medicine, Cairo University Children’s Hospital, Cairo, Egypt

**Keywords:** Maple-syrup-urine disease; Sanger sequencing, Exome sequencing, Novel variant, Egypt, Genetics, Molecular biology, Molecular medicine, Neurology

## Abstract

Maple Syrup Urine Disease (MSUD, OMIM# 248600) is an autosomal recessive inborn error of metabolism characterized by elevated branched chain amino acids (BCAA) leucine/isoleucine and valine in blood of affected children. The phenotypic and genotypic spectrum of MSUD is largely unreported in Egypt. We recruited ten patients (4 males/6 females, 2weeks-12years) from nine unrelated families with clinical and biochemical evidence of MSUD. We performed Sanger sequencing for the three most-commonly responsible genes: *BCKDHA*, *BCKDHB* and *DBT* and conducted exome sequencing for unresolved cases. Through Sanger sequencing, we detected eight homozygous pathogenic/likely pathogenic variants (four in *BCKDHB*, three in *BCKDHA* and one in *DBT* gene) in eight different families. The proband of family VI, who had no significant genetic findings by Sanger, had a peculiar phenotype and atypical radiological findings. His exome sequencing revealed a previously reported homozygous likely pathogenic variant in the *RARS2* gene (NM_020320.5:c.1026G > A;p.(Met342Ile)) causing the mitochondrial-encephalopathy disorder pontocerebellar hypoplasia, type 6 (OMIM# 611523). Furthermore, the copy-number-variant analysis of the exome data revealed a biallelic duplication affecting exons 2–6 of the *BCKDHB* gene (GRCh38: Chr.6-g.80127496:80171441dup) evaluated as variant of uncertain significance but expected to cause a breakpoint and may disrupt gene function, which can explain the markedly elevated BCAA levels in the patient’s blood. In conclusion, we expanded the genotypic and phenotypic spectrum of the disease and showed that aggressive intervention with specific treatment in the first few days of life resulted in normal development even in a developing country setting. Inclusion of MSUD in the national newborn screening program in Egypt is highly recommended.

**Supplementary Information:**

The online version contains supplementary material available at 10.1038/s41598-024-78105-y.

## Introduction

Branched-chain ketoaciduria or maple syrup urine disease (MSUD, OMIM#248600) is a rare autosomal recessive inborn error of branched chain amino acid (BCAAs; isoleucine, leucine, and valine) metabolism. It is caused by pathogenic variants in any of the main four different genes that encode for mitochondrial branched chain alpha ketoacid dehydrogenase complex (BCKD, EC 1.2.4.4)^1^. The BCKD complex is composed of 3 catalytic subunits, a heterotetrameric (alpha2/beta2) branched-chain alpha-keto acid decarboxylase; E1, coded by *BCKDHA* (OMIM* 608348) and *BCKDHB* (OMIM* 248611) genes, respectively, a homo-24-meric dihydrolipoyltransacylase; E2, coded by *DBT* (OMIM* 248610), and a homodimeric dihydrolipoamide dehydrogenase; E3, coded by *DLD* (OMIM* 238331)^2–4^. Pathogenic variants in these genes have been associated with MSUD^5^. This results in a diminished activity of the enzyme complex with the consequent accumulation of BCAAs with their corresponding branched chain ketoacids (BCKA) in cells and body fluids^2^. This manifests with wide scale of clinical phenotypes ranging from classical or severe phenotype with fatal neurometabolic symptoms typically in newborns to milder clinical phenotypes. These can present with ketoacidosis and mild neurological symptoms during infancy or early childhood. The neurotoxic manifestations of MSUD occur mainly due to high blood leucine and/or its keto acid level, α-keto isocaproic acid^6,7^.

Five clinical phenotypes of MSUD based on severity, age of onset, residual BCKD enzyme activity and thiamine responsiveness: classical, intermittent, intermediate, thiamine responsive and the E3 deficient that is associated with combined enzyme deficiencies in pyruvate dehydrogenase, α-ketoglutarate dehydrogenase, and BCKD, because E3 is a common component among these mitochondrial enzymes^8,9^. Classic or severe phenotype is the most common form and the patient commonly has less than 2% of normal enzyme activity^10^. It typically presents in neonatal period with maple syrup odor of cerumen shortly after birth and in urine within first week of life, poor feeding, vomiting, irritability, lethargy, focal dystonia, seizures, coma and central respiratory failure become evident by 4–10 days of life due to brain edema and progressive encephalopathy^11^. Both intermediate and intermittent phenotypes have milder clinical presentations and higher residual BCKD activity (3-30%)^10^. Patients may experience severe metabolic decompensation similar to the classic form during catabolic stress^12^.

Because of the severe acute onset of nonspecific clinical manifestations, MUSD can be clinically misdiagnosed as neonatal encephalopathy or neonatal sepsis. The earlier the initiation of specific treatment for MSUD, the higher the chance to reach normal milestones for mental and motor development. Therefore, MSUD is one of the earliest and most important inborn errors included in metabolic newborn screening programs all over the world^13^.

With a biochemical evidence of elevated BCAAs in blood, Sanger sequencing of the most commonly affected genes; *BCKDHA*,* BCKDHB* and *DBT* often reveals the genetic background of MSUD. Molecular diagnosis is also critical to confirm affected individuals and carriers of the disease, which is highly important for prenatal diagnosis and premarital genetic counseling in affected families^14^. However, few patients may require an extensive genetic technique, such as exome and genome sequencing to identify their genetic background.

The worldwide incidence of MSUD is approximately 1 in 185,000 with hot spots in certain ethnic groups, such as the Mennonite and Ashkenazi Jewish populations^15^. Although the prevalence of MSUD in the Arab population is not well studied, it is likely to be higher than most populations due to the autosomal recessive nature of the disease and the high prevalence of consanguineous marriages in the region. No data about the disease incidence is yet available in Egypt.

We conducted this study for investigating the genetic spectrum of 10 Egyptian pediatric patients with clinical features suggestive of MSUD and elevated BCAAs in blood spots detected by tandem mass spectrometry. We performed Sanger sequencing of the common causative genes *BCKDHA*,* BCKDHB* and *DBT* in all suspected individuals with their parents in a trial to confirm the variants segregation. Furthermore, we performed whole exome sequencing (WES) for case number 7 that didn’t reveal any causative variants by Sanger sequencing.

## Patients and methods

### Patients

We recruited ten clinically manifesting children belonging to nine unrelated families at the neurometabolic clinic, and neonatal ICU at Cairo University Children’s Hospital with suspected MSUD. Ethylenediamine tetra acetic acid (EDTA) blood samples were obtained from a total of 28 individuals (10 patients and 18 family members). MSUD patients were recruited based on family history of similar disease, suggestive clinical examination (poor feeding, vomiting, irritability, lethargy, seizures, coma and central respiratory failure and progressive encephalopathy) and elevated leucine/isoleucine and valine levels in blood spots as detected by mass spectrometric analysis. Patients were recruited over the period extending from January 2022 to June 2023 and all relevant data were collected from the patients’ records at Cairo University Children’s Hospital.

The study was conducted in accordance with the declaration of Helsinki for studies involving human participants and the study protocol was approved by the Research Ethics Committee at Faculty of Medicine, Cairo University, Egypt (Approval code #N-138-2021). Written informed consents were obtained from legal guardians of all participants prior to inclusion in the study.

### Biochemical analysis

Triple-quadruple tandem mass spectrometer (Quattro LC; Micromass, Waters, Manchester, UK) with a positive electrospray ionization probe using known concentrations of a mixture of isotopically labeled internal standards of amino acids and acylcarnitines, provided by Cambridge Isotope Laboratories (Woburn, MA, USA). Waters 2695 HPLC with an autosampler (Waters, Milford, MA, USA) was used for automatic injection. The extraction and analysis methods were as previously described^16^. Additional testing of urinary organic acid analysis was performed for selected patients by the Agilent 7890B GC with Agilent 7010 triple quadropole GC/MS and 7693 A autosampler (Agilent Technologies, Santa Clara, CA, USA) using non-labelled internal standards and following the methodology of La Marca and Rizzo, 2011^17^.

### Genomic DNA extraction, Sanger sequencing and exome sequencing

Whole blood was collected on EDTA vacutainer tubes for all suspected MSUD patients presenting to the neurometabolic clinic or admitted to the neonatal intensive care unit (NICU) at Cairo University Children’s Hospital and their parents. Genomic DNA was extracted using QIAamp^®^ DNA Mini and blood Mini Kit (QIAGEN, Hilden, Germany) according to manufacturer’s instructions. The quantity and quality of the extracted DNA were confirmed at wavelengths 260/280 and 260/230 by theNanoDrop2000 spectrophotometer (Thermo Fisher Scientific, Waltham, MA, USA). DNA was stored at − 20 °C until further processing.

All exons of the human *BCKDHA*,* BCKDHB* and *DBT* genes were amplified from the extracted genomic DNA of each index case using the specific primers designed by the investigators and listed in supplementary Tables 1–3. After identifying the causative variant in each index case, family segregation analysis was performed for the detected pathogenic variant in the corresponding exon for available family members. PCR reaction was prepared according to the standard protocol using Dream Taq Green PCR Master Mix (Thermo-Scientific, Waltham, MA, USA).PCR reactions were performed under a standard program with specific annealing temperature for each primer pair (the range was between 53.2 and 64.2 °C).

PCR products were separated on 2% agarose gel electrophoresis and then purified with Thermo Scientific™ GeneJET™ Genomic DNA Purification Kit (Applied biosystems, USA) according to manufacturer’s protocol. We stored the purified DNA at − 20 °C. The purified PCR products were sequenced using the BrillantDye^™^ terminator V3.1 Cycle Sequencing kit (NimaGen, the Netherlands) and the ABI 3500 gene analyzer.

For family VI, pathogenic variants were not revealed by Sanger sequencing, thus exome sequencing was performed. The coding and flanking intronic regions of interest were enriched using in solution hybridization technology and were sequenced using the Illumina, NovaSeq system. >99.9% of the targeted regions were covered by a minimum of 85 high-quality sequencing reads per base. Clean sequence reads were mapped to the human reference genome (GRCh37/hg19).

Human Genome Variation Society (HGVS) recommendations were used to describe sequence variants (http://www.hgvs.org). Variants were evaluated based on current scientific data and specific criteria according to the American College of Medical Genetics (ACMG) guidelines for evaluation of variant pathogenicity^18^. NGS based copy number variations (CNV) calling was computed based on uniquely mapped, non-duplicate, high quality reads and based on sequencing coverage depth. CNV calling was performed by computing the sample’s normalized coverage profile and its deviation from the expected coverage.

## Results

### Clinical features

**Fig. 1:**
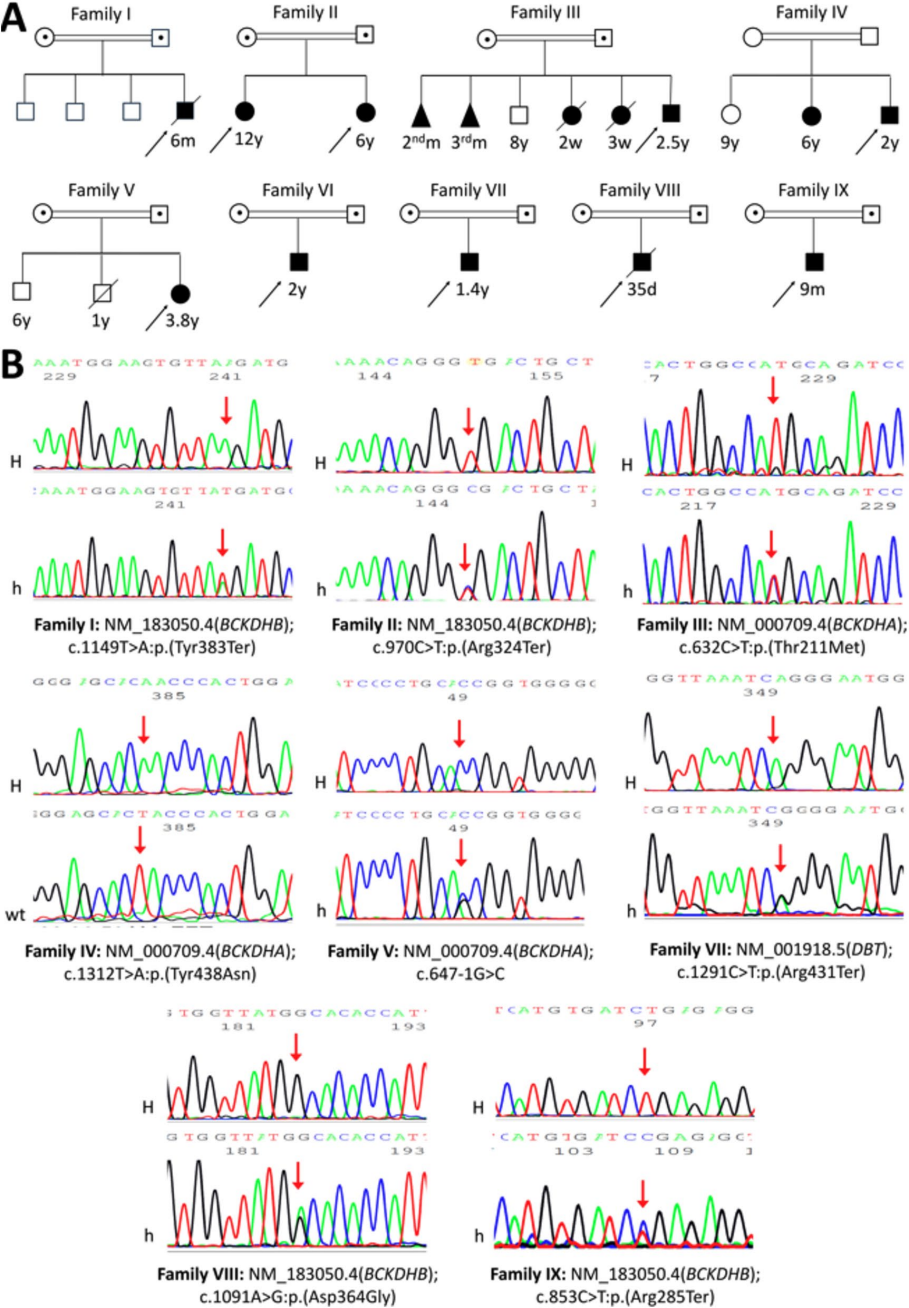
Family pedigrees and genetic variants in affected maple syrup urine disease (MSUD) patients. (**A**) Family pedigrees of all recruited children. (**B**) Detected genetic variants in *BCKDHA*, *BCKDHB* and *DBT* genes. All detected variants fully segregated in all tested family members (homozygous in the MSUD patients and heterozygous in their parents) consistent with the autosomal recessive inheritance nature of the disease. NM_number represents each gene transcript. *H* homozygous, *h* heterozygous, *A* adenine, *C* cytosine, *G* guanine, *T* thymine.

Ten Egyptian MSUD patients (4 males/6 females, age range 2 weeks-12 years) from nine unrelated families of Egyptian background were recruited (Including parents a total of 28 individuals were sampled). Figure 1A provides the family pedigrees of all recruited children. Consanguinity was reported in 100% of families. First-cousins marriages dominated our recruited parents except in families II, III and VI, in which they were second-cousins and family IV, which were more distantly related. Five families (56%) had history of previous sibling affection or previous unexplained death in early neonatal period. The demographic, clinical and biochemical features of affected patients are summarized in Table 1.

All ten recruited patients manifested during their first week of life after a short period of breast feeding with severe clinical features including poor suckling, vomiting, irritability, lethargy and moderate to severe epileptic fits. Maple syrup odor of urine was evident in all recruited newborns during their admission to the NICU. The amino acids leucine and valine were considerably elevated in the blood spots of all children; 2248 ± 1020 (Reference interval 0-270 µmol/l) and 439 ± 209 (0-290 µmol/l), respectively. Plasma ammonia levels were elevated in all investigated newborns at time of admission (8/8), while lactate levels were only elevated in a minority of patients (2/8). Due to the severe nature of the presenting features and the availability of specific diagnostic metabolic tests at our tertiary care facility, most patients were biochemically confirmed by either amino acid analysis by tandem mass spectrometry or urinary organic acid analysis by gas chromatography mass spectrometry within the first month of their lives.

**Table 1:** Demographic, clinical and biochemical features of Egyptian maple syrup urine disease (MSUD) patients (*n* = 10).

Family/Patient code	Gender	Current age	Age at diagnosis	Consanguinity	Family history	Clinical neurometabolic manifestations						Relevant investigations at diagnosis					
						Poor suckling & Vomiting	Lethargy/irritability	Maple syrup odor	Seizures	Coma	phenotype	Leucine/ isoleucine (µmol/l)	Valine(µmol/l)	Leucine/Phenylalanine	Urine organic acids	Ammonia (µmol/l)	Lactate (mg/dl)
I/1	M	Died at 6 m	1 m	1st cousins	One Previous abortion	Yes (7d)	Yes (7d)	Yes	Yes	–	classic	2164	311	29.9	2OH butyric acid, 2-ketoglutraric acid	128	11
II/2	F	12y	1 m	2nd cousins	–	Yes (2d)	Yes (3d)	Yes (7d)	Yes (7d)	Yes (7d)	classic	1482	300	17.5	NA	237	15.2
II/3	F	6y	3d	2nd cousins	MSUD female sibling	–	–	Yes (15d)	–	–	mild	512	106	8.5	NA	NA	NA
III/4	M	2.5y	15d	1st cousins	Two previous abortions & two siblings died at 14d	–	–	Yes (7d)	Yes (4d)	Yes (4d)	classic	3252	893	11.3	NAD	73	9.8
IV/5	M	2y	13d	3rd degree	MSUD female sibling	Yes (7d)	Yes (7d)	Yes (15d)	Yes (7d)	–	classic	884	362	15	NA	163	13
V/6	F	3.8y	37d	1st cousins	Male sibling died at 1y	Yes (4d)	–	Yes (1 m)	Yes (4d)	–	classic	2478	500	NA	Lactate,2-OH isovaleric, 2- oxocaproic acid	NA	NA
VI/7	M	2y	18d	2nd cousins	–	Yes (5d)	Yes (5d)	Yes (1 m)	Yes (9d)	Yes (9d)	atypical	3590	405	NA	4-OH Phenyl lactate, 4-OH phenyl Pyruvate	78.2	12.3
VII/8	F	1.4y	1 m	1st cousins	–	–	Yes (4d)	Yes (1 m)	Yes (4d)	Yes (1 m)	classic	3160	432	49	NA	183	29
VIII/9	F	Died at 35d	15d	1st cousins	–	Yes (9d)	Yes (9d)	Yes	Yes (12d)	Yes (12d)	classic	2696	505	56.8	Lactate,2-OH isovaleric,2-oxocaproic acid	206	35.2
IX/10	F	9 m	17d	1st cousins	–	Yes (3d)	Yes (3d)	Yes (3d)	Yes (13d)	-----	classic	2260	574	33.7	NA	135	10.2

*d* day, *F* female, *M* male, *m* month, *NA* data not available, *NAD* no abnormality detected, *NBS* newborn screening, *y* year, Reference interval^19^: Leucine/isoleucine (0-270) µmol/l; Leucine/phenylalanine ratio (0–5) µmol/l; Valine (0-290) µmol/l; ammonia (11–51)µmol/l; lactate (4.5–19.5) mg/dl.

The majority of recruited children at time of genetic confirmation had the classic phenotype of MSUD, with delayed mental and motor development and recurrent episodic attacks of decompensation upon exposure to a stressful condition, such as infections and intercurrent illnesses. Only a single patient (Patient II/3) had a milder course. She was diagnosed at 3 days of age due to a previous confirmed sibling in the family and started receiving the specific MSUD formula immediately after diagnosis with a very good compliance from the family. This child at 6 years of age had almost normal motor and mental development and only suffered few attacks of mild metabolic decompensation.

One of our recruited children (proband V/6) suffered from severe leucine/isoleucine and valine deficiency during treatment leading to the dermatological manifestations of acrodermatitis dysmetabolica. Increasing the isoleucine and valine intake in her diet improved the inflammatory skin manifestations and alopecia.

Another peculiar patient (proband VI/7) although having a very high leucine level initially (3590 µmol/l), he responded very well to early specific diet and peritoneal dialysis therapy followed by early specific diet and his follow-up levels were mostly controlled (< 500 µM) apart from a few elevations during decompensation episodes. However, clinically he showed marked developmental delay and progressive microcephaly that slowed down after the first year of life. Although brain MRI usually shows characteristic MSUD features that can be interpreted by an expert pediatric neuro-radiologist irrespective of the clinical and laboratory data^20^, the recent brain MRI of proband VI/7 at 23 months of age revealed atypical signs of atrophic brain changes and bilateral rather symmetrical signal alteration in both basal ganglia particularly the putamen, present as marked atrophy and seen replaced by cystic encephalomalacia. Prominent extra axial CSF space and basal cisterns, corpus callosum thinning and cerebellar vermian hypoplasia were also observed (Supplementary Fig. 1). Individual clinical summaries of all recruited children are further provided in Supplementary Data file.

### Genetic diagnosis

Sanger sequencing of *BCKDHA*, *BCKDHB* and *DBT* genes conducted for the nine affected families revealed eight different homozygous pathogenic/likely pathogenic variants (four in *BCKDHB*, three in *BCKDHA* and one in *DBT* gene). The variants were four nonsense, three missense and one splice-acceptor variant. All eight detected variants by Sanger sequencing were previously reported. In the *BCKDHB* gene, we detected c.1149T > A;p.(Tyr383Ter), c.853 C > T;p.(Arg285Ter)^21^, c.970 C > T;p.(Arg324Ter)^22,23^ and c.1091 A > G;p.(Asp364Gly)^24^. While in *BCKDHA* gene, we detected c.632 C > T;p.(Thr211Met)^25^, c.1312T > A;p.(Tyr438Asn)^21^ and c.647-1G > C^3^. As for *DBT* gene, we detected a single variant c.1291 C > T;p.(Arg431Ter)^26^. All variants were fully segregated in affected family members as all affected children were homozygous and all parents were heterozygous consistent with the autosomal recessive inheritance of the disease (Fig. 1B).

In family VI (Proband 7), Sanger sequencing didn’t detect any pathogenic/likely pathogenic variants in the three genes, thus an extensive sequencing technique (exome sequencing) was performed for the proband of this family in a trial to reveal his genotype. Short variant analysis didn’t reveal any variants in either of *BCKDHA*,* BCKDHB*,* DBT* or *DLD* genes supporting the Sanger sequencing analysis; however, a previously reported homozygous likely pathogenic variant was detected in the *RARS2* gene (NM_020320.5:c.1026G > A;p.(Met342Ile))^27^ causing the mitochondrial encephalopathy disorder pontocerebellar hypoplasia, type 6 (OMIM# 611523), whose clinical phenotype overlaps with MSUD. Furthermore, the CNV analysis of the exome data of the proband revealed a duplication affecting exons 2–6 of the *BCKDHB* gene (GRCh38: Chr6-g.80127496:80171441dup). The variant is novel, absent in population databases and evaluated as variant of uncertain significance (VUS) according to the ACMG criteria; however, is expected to cause a breakpoint in the gene transcript that may disrupt its function. Table 2 summarizes all detected variants in the nine recruited families.

**Table 2:** Disease causing variants detected in Egyptian children with maple syrup urine disease (MSUD) (*n* = 10).

Family	Patient	Chromosomal location (GRCh38)	Gene: Nucleotide change*	Exon/Intron	Protein effect*/ Zygosity	Zygosity	Variant effect	rsID	ACMG classification	ClinVar	MAF exomes/ genomes	References
I	1	Chr6-80343774-T-A	*BCKDHB*:c.1149T > A	Exon 10	p.(Tyr383Ter)	H	Nonsense	rs190867671	Pathogenic	P/LP	0.00/ 0.00	Henneke et al., 2003
II	2,3	Chr6-80273153-C-T	*BCKDHB*:c.970 C > T	Exon 9	p.(Arg324Ter)	H	Nonsense	rs398124603	Pathogenic	P/LP	0.0000598/ 0.0000132	McConnell et al., 1997; Puckett et al.,2010
III	4	Chr19-41419282-C-T	*BCKDHA*:c.632 C > T	Exon 5	p.(Thr211Met)	H	Missense	rs398123503	Pathogenic	P/LP	0.000024/ 0.00000657	Georgiou et al.,2009
IV	5	Chr19-41424582-T-A	*BCKDHA*:c.1312T > A	Exon 9	p.(Tyr438Asn)	H	Missense	rs137852870	Pathogenic	P/LP	0.0000643/ 0.000118	Zhang et al.,1989; Henneke et al., 2003
V	6	Chr19-41422163-G-C	*BCKDHA*:c.647-1G > C	Intron 5–6	-----	H	Splicing	rs753216964	Pathogenic	P	0.00/ 0.00000657	Jaradat et al.,2015
VI	7	Chr6-80127496-80171441dup	*BCKDHB*	Exons 2–6 duplication	-----	H	CNV	-----	VUS	-----	0.00/ 0.00	This study
		Chr6-87521473-C-T	*RARS2*:c.1026G > A	Exon 12	p.(Met342lle)	H	Missense	rs34647222	Likely Pathogenic	P/LP/ VUS	0.000255/ 0.000158	Pronicka et al.,2016
VII	8	Chr1-100196413-G-A	*DBT*:c.1291 C > T	Exon 11	p.(Arg431Ter)	H	Nonsense	rs398123660	Pathogenic	P/LP	0.00000843/ 0.0000187	Sun et al.,2020
VIII	9	Chr6-80343716-A-G	*BCKDHB*:c.1091 A > G	Exon 10	p.(Asp364Gly)	H	Missense	-----	Likely Pathogenic	-----	0.00/ 0.00	Khalifa et al., 2020
IX	10	Chr6-80203114-C-T	*BCKDHB*:c.853 C > T	Exon 8	p.(Arg285Ter)	H	Nonsense	rs398124598	Pathogenic	P/LP	0.0000137/ 0.000145	Henneke et al., 2003

*Variants nomenclature is according to *BCKDHA*: NM_000709.4 and NP_000700.1; *BCKDHB*: NM_183050.4 and NP_898871.1; *DBT*: NM_001918.5 and NP_001909.4; *RARS2*: NM_020320.4 and NP_064716.2 Refseq gene transcript and protein, respectively; MAF, minor allele frequency according to gnomAD population database (https://gnomad.broadinstitute.org/); H, homozygous. All variants segregated properly in both parents of the diseased children according to strict autosomal recessive inheritance.

## Discussion

In the current study, we evaluated the clinical, biochemical and genetic fingerprints of ten MSUD patients from nine unrelated Egyptian families. All patients were offspring of consanguineous parents emphasizing the importance of prenatal diagnosis and proper premarital genetic counseling in families with history of the disease.

MSUD is a rare inherited metabolic disorder and its prevalence and genetic makeup are not fully identified in Egypt nor in other Arab populations. We investigated the MSUD database from Centre for Arab Genomic Studies (CTGA database ) [https://cags.org.ae/en/ctga-details/199/maple-syrup-urine-disease*]* and found only a limited number of patients that were reported mainly from Lebanon, UAE, Jordan, Sudan, Egypt, Oman, Kuwait, Qatar and Saudi Arabia^24,28–37^.

In Egypt, we suffer a great lack of awareness of general practitioners concerning inherited disorders of inborn errors of metabolism especially in rural areas. Furthermore, the lack of a newborn screening program for inherited metabolic disorders leads to the delay in diagnosis and the development of severe and irreversible complications that result in handicapped children and sometimes fatality before diagnosis. We conducted this study to raise the awareness of health care physicians about MSUD by highlighting possible preliminary screening and diagnostic strategies. Moreover, identifying the underlying genetic background of MSUD is essential for proper genetic counseling and prenatal diagnosis to minimize the incidence of the disease. Recently the presidential health-care initiative that started in 2021 to screen diseased newborns and infants in governmental ICUs allover Egypt for metabolic disorders will provide an initial step to identify and manage such inherited diseases at earlier stages.

In our study, all recruited patients were confirmed to have a high level of BCAAs by LC-MS/MS at a mean age of 20.1 ± 9.4 days (range 3-37days) (Table 1). The delay in the diagnosis can be attributed to nonspecific initial manifestations simulating neonatal sepsis with absence of family history of MSUD. Thus, highlighting the need for the regular implementation of newborn screening program by tandem mass spectrometry to detect presymptomatic cases, which will give a better chance for early specific management for MSUD affected newborns avoiding a potentially fatal outcome and improving their motor and mental development^5,38–40^.

The clinical outcome of MSUD is usually remarkable if it is detected early either through routine newborn screening or prenatally in affected families. Considerable elevations of BCAAs can be detected 48–72 h after normal breast feeding through tandem mass spectrometry of dried blood spots^41^. Considerable elevations of BCAAs commonly accompanied by reduced other amino acids, leading to increased concentration ratios such as leucine/phenylalanine ratio can be detected 48–72 h after birth through tandem mass spectrometry in dried blood spots. Moreover, GC-MS analysis of urine organic acid profile of positive cases can detect branched chain keto acids (BCKAs) that correlate with the elevation of BCAAs, such as α-ketoisocaproic and α-keto-β-methyl isovaleric acids. This can give a very good diagnostic evidence of MSUD. Furthermore, genetic testing of the causative genes by Sanger sequencing or NGS is crucial to detect the causative variant for detecting presymptomatic familial cases, prenatal diagnosis and genetic counseling of affected families^41^.

The treatment strategy of MSUD is mainly directed towards lowering the plasma level of BCAAs to prevent acute and chronic sequalae of the disease. In the acute state, hemodialysis or peritoneal dialysis is the most effective mode to rapidly washout BCAAs from blood and tissues usually within 24 h. MSUD patients must remain on artificial formulas of protein-restricted diet lacking leucine, isoleucine and valine. However, these three amino acids are essential, so they are added to the diet separately in small amounts depending on their plasma levels to support for normal development and ensure that the patient’s condition and biochemistry remain within an acceptable therapeutic range (https://rarediseases.org/rare-diseases/maple-syrup-urine-disease/#diagnosis). Therefore, patient II/3, the sister of proband (II/2) was picked up and diagnosed at the age of 3 days before symptomatizing and standard MSUD formula was started earlier with close follow up. So, this child at 6 years shows normal developmental growth for age. However, she experiences occasional mild metabolic decompensation during intercurrent illnesses. Based on this sibling, the published literature^38–40^ and our previous experience with other early treated neonates of MSUD, the ideal age to start treatment is the newborn screening age (2–7 days). Other factors, such as availability of the special formula and family compliance also play a significant role in the therapeutic response and the incidence of complications; however, starting treatment during the first week of life is the strongest factor.

We identified eight different homozygous pathogenic/likely pathogenic fully segregating variants under strict autosomal recessive conditions and reported in several populations as disease causing as shown in Table 2. Half of the variants identified in our study were in *BCKDHB* gene, followed by *BCKDHA* gene (3 families) and *DBT* gene (1 family), which is matching the distribution of pathogenic variants over the three genes as reported in several studies^21,42–44^.

In *BCKDHB* gene, we identified three nonsense variants: c.1149T > A; p.(Tyr383Ter) detected in family I, reported in Turkish patients with classic MSUD^21^, c.970 C > T; p.(Arg324Ter) detected in family II, reported in USA^22,23^, India^45^ and Egypt^24^ and c.853 C > T;p.(Arg285Ter) detected in family IX reported in Turkey^21^, Spain^4^, Portugal^46^, India^45^, Saudi Arabia^39^ and China^47^. Moreover, a missense variant c.1091 A > G; p.(Asp364Gly) detected in family VIII in our study. This variant replaced the negatively charged aspartate at position 364 with the neutral glycine disturbing the rigidity of E1B molecule and leading to the failure of hydrogen bond formation at this location. The variant has been only reported in a single Egyptian MSUD family^24^.

We further identified a potentially novel homozygous duplication affecting exons 2–6 of the *BCKDHB* gene (GRCh38: g.80127496:80171441dup) that was encountered in family VI. This variant is not reported in ClinVar and evaluated as VUS, according to the ACMG criteria for the classification of pathogenic variants. It is also completely absent in population databases. This duplication should affect thiamine diphosphate binding domain and the carboxyl domain of E1 beta protein, which both play crucial role in its enzymatic activity. Whereas CNV deletions commonly lead to loss-of-function, duplications might cause disease through several mechanisms including triplosensitivity, gene disruption, or gene fusion at breakpoints^48^. Since the protein encoded by the *BCKDHB* gene is an established loss-of-function protein, the potential harmful effect of this duplication CNV variant is most probably through the disruption of gene function by affecting a splice site leading to exon skipping and the production of a nonfunctional protein. Unfortunately, an mRNA sample was not available for our proband, thus investigating the variant at the cDNA level was not feasible. Although large duplications are not commonly reported as a cause of disease for MSUD in the literature, a similar duplication affecting exons 7–9 in *BCKDHB* has been reported as likely pathogenic for MSUD in ClinVar on 2 separate occasions: (SCV000931442 and SCV002556306).

In the same proband (family VI/7), a homozygous likely pathogenic previously reported variant was detected in the *RARS2* gene. The *RARS2* (Arginyl-tRNA synthetase 2) gene (OMIM* 611542) encodes a protein that localizes to the mitochondria, where it catalyzes the transfer of L-arginine to its cognate tRNA, an important step in translation of all mitochondrially-encoded proteins. Biallelic variants in the *RARS2* gene are reported to cause a mitochondrial encephalopathy (Pontocerebellar hypoplasia, type 6 (PCH 6); OMIM# 611523). Characteristic clinical features comprise neonatal severe encephalopathy, intractable seizures, feeding problems lactic acidosis, and profound developmental delay^49^. In affected patients, MRI commonly reveals hypoplasia of pons and cerebellum, followed by general brain atrophy^50^. However, clinical heterogenicity of the syndrome has been reported in several studies in which the typical neuroradiological findings of cerebellar hypoplasia and progressive pontocerebellar atrophy may be absent in some affected patients^51–53^.

The homozygous missense variant in exon-12 of the *RARS2* gene in our proband, NM_020320.5:c.1026G > A;p.(Met342Ile) is evaluated as likely pathogenic, according to the ACMG criteria. The variant is absent in gnomAD and has been reported as disease causing in several patients with mitochondrial encephalopathic features^27,54,55^. MRI findings in our proband didn’t show the characteristic pontocerebellar hypoplasia; however, the MRI features were not typical of MSUD as well. Furthermore, the *RARS2* variant cannot explain the repeatedly elevated leucine/isoleucine levels in the patient blood. Hence, we believe that the severe and atypical presentation of this proband is due to a dual genetic background affecting both *BCKDHB* and *RARS2* genes.

Three previously reported homozygous fully segregating pathogenic variants in *BCKDHA* gene (two missense and one splice-acceptor) were also detected. The missense variant c.632 C > T;p.(Thr211Met) detected in family III, was previously reported in Greek and Cyprus patients^25^. A substitution of threonine by methionine disturbs the access of the potassium ion to the potassium binding pocket of the E1α subunit resulting in a collapse of the potassium-binding pocket and absence of bound thiamine diphosphate (ThDP) cofactor by the mutant protein, therefore, absence of overall BCKD complex activity^25^. The second missense variant was c.1312T > A;p.(Tyr438Asn), detected in family IV, was first identified as Tyr394Asn in the NIGMS Human Genetic Mutant Cell Repository in USA^56^, then correctly identified and described as Tyr438Asn in German and Egyptian patients^21,24^. Finally, the splicing variant c.647-1G > C identified in family V was previously reported in Jordanian patients^3^ and Cypriotic patients^25^ associated with classical MSUD.

**Fig. 2:**
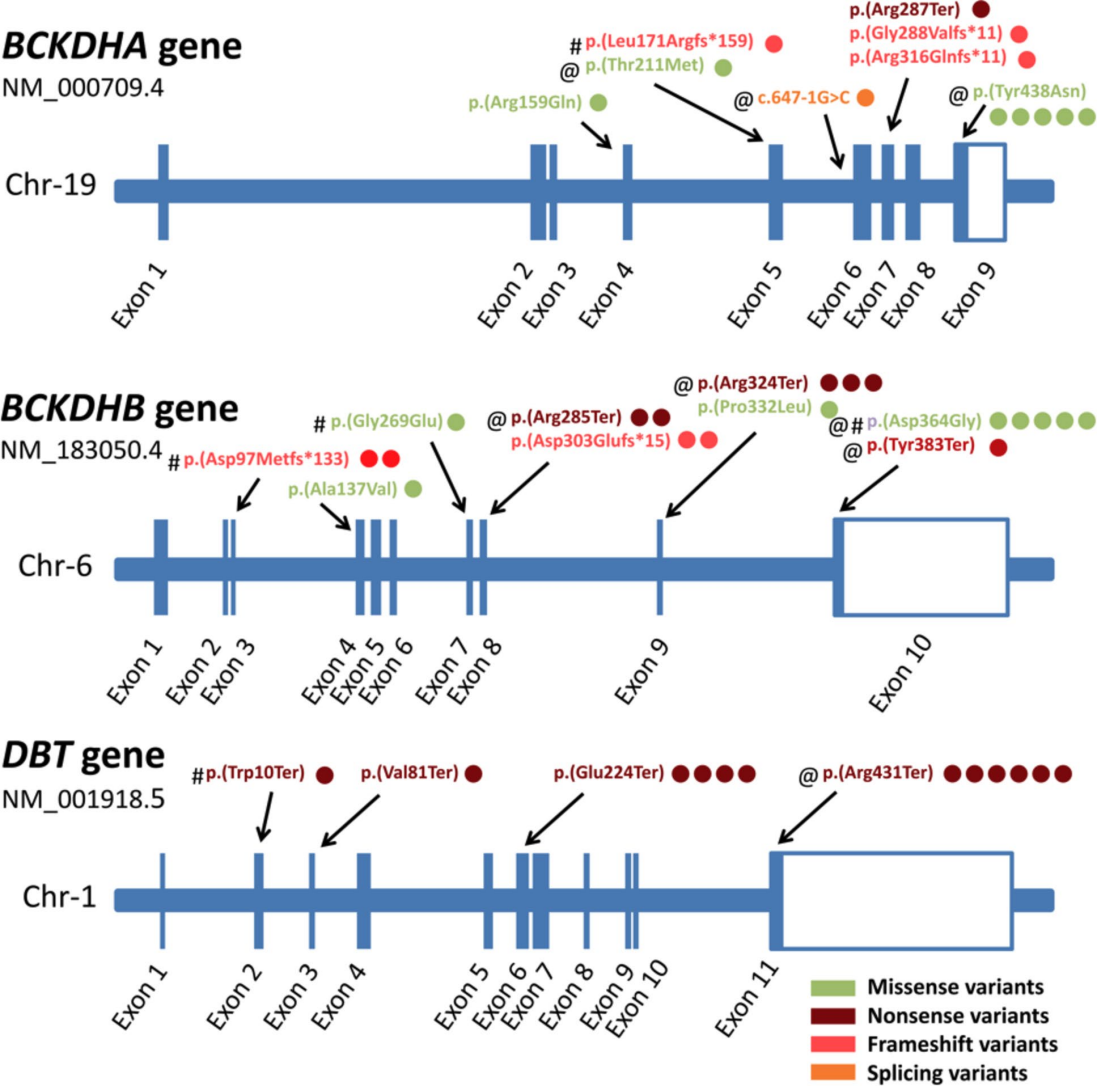
All reported variants in this study and the literature in Egyptian MSUD patients with their familial frequencies. The number of families affected by each variant is represented by the number of circles adjacent to each variant based on the variants reported by Khalifa et al., 2020; Dahpy et al., 2021 and this study. The symbol @ represents variants reported in our study and # represents variants reported only in Egyptian patients and absent in the literature or international MSUD databases so far.

A single pathogenic nonsense variant c.1291 C > T;p.(Arg431Ter) has been identified in family VII and was previously reported in Chinese patients. This nonsense variant results in truncated protein with the loss of the 2-oxoacid dehydrogenase acyltransferase catalytic domain^26^. Five of our detected variants in this study have been previously reported in Egyptian MSUD patients by Khalifa et al., 2020^24^; however, four variants were not reported in Egyptian population including the novel variant in *BCKDHB* (exons 2–6 duplication). Figure 2 demonstrates all the variants reported in Egyptian MSUD patients in this study and previous ones^24,57^, with the number of affected families reported with each variant. Five variants overall are only reported in Egyptian population so far.

In conclusion, genetic testing of causative genes in MSUD is crucial to confirm the diagnosis and provide proper genetic counseling and prenatal diagnosis for the affected families. We expanded the genotypic spectrum of the disease with a novel CNV duplication that may cause disruption of the protein function. Although the available data is not conclusive regarding the pathogenicity of this variant in absence of an RNA evidence, it is the most likely explanation for the elevated levels of branched chain amino acid in the proband and thus it may contribute to his phenotype. We further highlighted a patient with a rare complication of MSUD treatment leading to severe dermatological features of acrodermatitis dysmetabolica. In our study, we didn’t observe a clear phenotype-genotype correlation in our patients as the majority had a severe phenotype; however, early and aggressive intervention with specific treatment in the first few days of life resulted in normal development and a reasonable chance of a healthy productive life style as the second affected sibling in family II in our study, thus similar to other nations a newborn screening program for MSUD is crucial especially that Egypt has high rates of consanguineous marriages paving the way for a higher incidence of recessive genetic disorders.

## Electronic supplementary material

Below is the link to the electronic supplementary material.

Supplementary Material 1

## Data Availability

All data generated or analyzed during this study are included in the published article (and its supplementary information files). Any further clinical or genetic data are available from the corresponding authors upon reasonable request. The novel variant reported in this study has been submitted to the Leiden Open Variation Database (LOVD) under variant submission code: #0000957952.
