# Supplementary material for: Expanding the genotypic and phenotypic spectrum of Egyptian children with maple syrup urine disease
Source: Sci Rep. 2024 Nov 18;14:28391. doi: 10.1038/s41598-024-78105-y (PMC11570613; doi:10.1038/s41598-024-78105-y)
Supplement: Supplementary file 1 — Supplementary Material 1 [file 41598_2024_78105_MOESM1_ESM.pdf]

Supplementary data

Expanding the genotypic and phenotypic spectrum of Egyptian children with maple syrup urine disease

Zeinab S. Abdelkhalek[1], Shadia M. Hussein[2], Iman G. Mahmoud[3], Areef Ramadan[3], Mona A. Kamel[3], Marian Y. Girgis[3], Mohamed A. Elmonem[1]

[1] Clinical and Chemical Pathology Department, Faculty of Medicine, Cairo University, Cairo, Egypt

[2] Central laboratories, Cairo University Hospitals, Cairo University, Cairo, Egypt

[3] Pediatric Neurology Department, Metabolic Division, Faculty of Medicine, Cairo University Children's Hospital, Cairo, Egypt

Detailed clinical data of recruited families:

Family I:

It consists of four male siblings and their parents and the family originates from Giza governorate, Egypt. The proband (I/1)was male (youngest one in order of his siblings). The mother had history of three previous abortions. The proband manifested at 7 days of age by poor suckling, disturbed conscious level and seizures,Subsequently, he was admitted to the NICU with a suspicion of neonatal sepsis or encephalopathy but without improvement on recommended treatment regimens.Therefore,at 30 days, metabolic screening by mass spectrometry and MRI brain were ordered. The MRI revealed abnormal white matter signal intensity at the bilateral cerebellar and cerebral peduncles, posterior limb of internal capsule, perirolandic cerebral white matter, periventricular and dorsum brain stem, eliciting low T1, high T2/FLAIR signal intensity, diffusion restriction limited to the posterior limb of the internal capsule, the dorsum brain stem as well as the optic radiations. The metabolic screening revealed the diagnosis of MSUD. The standard MSUD formula was prescribed with regular follow up with TMS. However, the newborn experienced marked neurometabolic decompensation during intercurrent illness and died at the age of 6 month.

Family II:

It consists of two affected female siblings and their parents and the family originates from Suez governorate, Egypt. The proband (II/2) was diagnosed with MSUD since she was one-month-old, when she suffered from poor suckling, vomiting at the age of 3 days. Maple syrup odor of cerumen and urine appeared later and subsequent convulsions and cardiac arrest at the age of 7 days, while she was admitted to NICU

at Cairo University Hospitals. She was confirmed as MSUD at the age of 1 month by metabolic screening; however, specific treatment was delayed. Currently, she is 12-years-old and has significant delayed motor and mental development. Her sister (II/3), on the other hand, was picked up and diagnosed at the age of 3 days, so standard MSUD formula was started earlier with close follow up. Therefore, this childat 6 years shows normal developmental growth for age. However, she experiences occasional mild metabolic decompensation during intercurrent illnesses.

Family III:

It consists of two male siblings and their parents. The mother had positive history of previous two abortions and the death of two female siblings at the age of two weeks, the family originates from Giza governorate, Egypt. The proband (III/4) is a male.He suffered from coma, convulsions and respiratory distress at 4 days of age,only after 3 days of breast feeding. He was admitted to the NICU and was later diagnosed at the 17th day by TMS metabolic screening. Brain MRI showed characteristic MSUD features; however, flash visual evoked potential (VEP) showed functionalvisual pathways. The patient at 2.5 years has delayed motor milestones with axial hypotonia, peripheral hypertonia and hyperreflexia in addition to delayed mental development.

Family IV:

It consists of three siblings (two females and one male) and 3rd degree consanguineous parents. The family originates from central Egypt. A previous female sibling diagnosed as MSUD in neonatal period and controlled on MSUD formula was unavailable for sampling. The proband (IV/5) was a male and was confirmed in utero to have Fallot's tetralogy by four-dimensional ultrasonography. At the age of 7 days,

he suffered from poor feeding, vomiting and irritability after breast feeding. Consequently, he developed convulsions for which he was admitted at NICU and metabolic screening was performed at two weeks of age and he was confirmed as MSUD. He received the MSUD formula feeding and underwent peritoneal dialysis in attempt to lower his branched-chain amino acids levels.

Family V:

It consists of two siblings (one male and one female) and first-degree consanguineous parents. The family had positive history of previous sibling death at the age of one year but was probably not related to MSUD. The family originates from Al-Qanater, Qalyubia Governorate, Egypt. The proband (V/6) is a female child. She developed convulsions and sepsis like picture at the age of 4 days after birth and was admitted to the NICU for 37 days where she was diagnosed as MSUD by TMS, urine organic acids and MRI findings of MSUD. She experienced skin manifestations of acrodermatitis dysmetabolica after a period of poor MSUD formula intake. She was detected to have low isoleucine and normal zinc levels. Increasing the leucine dose improved the skin manifestations and alopecia, thus the diagnosis of acrodermatitis dysmetabolica secondary to isoleucine deficiency was made. This case highlights the importance of cautiously balancing BCAAs levels while treating MSUD, as deficiency can trigger acrodermatitis dysmetabolica.

Family VI:

It consists of one sibling and his consanguineous parents. They originate from Fayoum governorate, central Egypt. The proband (VI/7) is a male child who was a full-term neonate with uncomplicated vaginal delivery and unremarkable family

history of metabolic disease. At day 5, he suffered from poor suckling, vomiting, irritability and lethargy so he was admitted to the NICU at 8 days of age. He developed convulsions with respiratory distress and was mechanically ventilated. Antibiotic regimen for presumptive neonatal sepsis was prescribed but without improvement. Initial biochemical investigations showed metabolic acidosis and a mild elevated ammonia level. Brain imaging showed hypoxic ischemic insult affecting mainly basal ganglia and ventricular and basal ganglia dilation and paraventricular hypodense areas.Further biochemical analysis by expanded metabolic screening at 18 days of age revealed marked elevated Leucine and valine in blood and nonspecific elevation of 4-OH phenyl lactate and 4-OH phenyl pyruvate in urine organic acid analysis.

Since then, the patient underwent peritoneal dialysis 3 times and received the MSUD formula regularly. This was followed by improvement of blood leucine level and conscious level and the patient was extubated and discharged. He experienced several incidences of metabolic decompensations with high level of BCAAs and admission in NICU during catabolic stress. Brain MRI was performed at 23 months, revealed signs of atrophic brain changes in the form of bilateral rather symmetrical signal alteration in both basal ganglia particularly the putamen. No perifocal edema or significant diffusion restriction were observed. Bilateral periventricular symmetrical abnormal signal was seen bright in FLAIR associated with sharp prominent lateral ventricles walls. Prominent extra axial CSF space and basal cisterns, corpus callosum thinning and cerebellar vermian hypoplasia were also observed **(Supplementary Figure 1)**. Currently he developed progressive microcephaly that became stationary at a later stage; initially he had a head circumference SD of -0.44 at two months of age and progressed to -2.3 at the age of 10 months and -2.5 at 23 months old. Currently, he

also has axial hypotonia with peripheral hypertonia, hyperreflexia and mild bulbar weakness.

Family VII:

It consists of a female proband and her parents. The family originates from Beni-Suef governorate in Central Egypt. The disease started in the proband (VII/8) at 4 days of age by lethargy and generalized tonic clonic seizures. She got admitted to the NICU, where she was mechanically ventilated. Initial laboratory tests showed metabolic acidosis, high blood ammonia and lactate but normal blood glucose. Metabolic screening was performed at one month of age and showed marked elevation of leucine/isoleucine and valine levels. Thus, the diagnosis of MSUD was established based on clinical and laboratory data. The patient received MSUD treatment regimen with improvement of her conscious levels.

Family VIII:

It consists of the female proband (VIII/9) and her consanguineous parents. They originate from Tanta city, Gharbia governorate, Egypt. The proband was a full-term neonate with normal CS delivery and unremarkable family history. The condition started at day 9 with poor suckling, irritability and lethargy. Initial blood tests revealed normal anion gap metabolic acidosis with high ammonia and lactate levels. She was admitted in NICU and later developed tonic clonic seizures, coma, apnea and was mechanically ventilated. Metabolic screening by LC-MS/MS and urine organic acids was performed at two weeks of age and the diagnosis of MSUD was established. The patient underwent peritoneal dialysis; however, she succumbed to her illness at the age of 35 days.

Family IX:

It consists of one female proband (IX/10) and her consanguineous parents. The family originates from Fayoum governorate, central Egypt. She manifested at 3 days with poor suckling and irritability that gradually progressed to convulsions at 13 days. She was admitted to the NICU and initial laboratory tests showed normal anion gap metabolic acidosis and high ammonia. Metabolic screening by mass spectrometry revealed marked elevated BCAAs. Therefore, the patient received MSUD treatment regimen with anticonvulsant control of seizures and discharged with close follow up of BCAAs level. The patient experienced several episodes of metabolic decompensation.

Supplementary Tables:

Supplementary Table1: PCR primers for genomic amplification of the human *BCKDHA* exons(5’ → 3’).

Primer location	Primer Name	Primer sequence	Number of base	Annealing temperture	Amplicon size (bp)
**Exon 1**	BCKDHA_EX1_F	CTGGTCAGGTTGCCCTCTT	19	64.2	421
	BCKDHA_EX1_R	GGACCCCACACTCTGAAGATAG	22		
**Exon2-3**	BCKDHA_EX2-3_F	CACATGCTCAACCACCATG	19	57.6	572
	BCKDHA_EX2-3_R	AATCTGTCCTCCTTGGGA	18		
**Exon4**	BCKDHA_EX4_F	GGGCTTCTATCACAGCAACT	20	61	402
	BCKDHA_EX4_R	CTCCTGGAAGAACACTCAGA	20		
**Exon5**	BCKDHA_EX5_F	TTTCCTGTCTGCCTGCCA	18	62.3	406
	BCKDHA_EX5_R	GAAGAAGAAGTCTGGAGCACAG	22		
**Exon6**	BCKDHA_EX6_F	GTGGGTCATGTGAGTGTGAATG	22	59.1	391
	BCKDHA_EX6_R	ACAGGACGAGAACCAGGAAG	20		
**Exon7**	BCKDHA_EX7_F	AGTTGAGGTCCTGAGCAC	18	62.5	385
	BCKDHA_EX7_R	TAAAGGCAAGGGGGAGATG	19		
**Exon8**	BCKDHA_EX8_F	TGACAGCCACCGTAGCAT	18	61	460
	BCKDHA_EX8_R	TATCCCTGAGCCTCCCAGTT	20		
**Exon9**	BCKDHA_EX9_F	CTTCTCTGTGCCTCAGTTTCCTC	23	62.5	599
	BCKDHA_EX9_R	AGGAGCAGGGGTGAAGAGT	19		

Supplementary Table 2: PCR primers for genomic amplification of the human*BCKDHB* exons (5’ → 3’).

Primer location	Primer Name	Primer sequence	Number of base	Annealing temperture	Amplicon size (bp)
**Exon 1**	BCKDHB_EX1_F	CCCAGAAACTATTTCATTGGC	21	59.5	565
	BCKDHB_EX1_R	CCAGAGAGTCAGGAAGTTAA	20		
**Exon2**	BCKDHB_EX2_F	CTCCAGGTCTGTATTGCTTTT	21	59.5	383
	BCKDHB_EX2_R	GCCCCAATCATACCTTTGAA	20		
**Exon3**	BCKDHB_EX3_F	GCTGCTATGTATCTACAGTGC	21	63.7	640
	BCKDHB_EX3_R	CAACAGGCAGAATCTCCAA	19		
**Exon4**	BCKDHB_EX4_F	CCTCTACCTGTTCTATACTTCTCC	24	59.6	444
	BCKDHB_EX4_R	ACCCAATCCCAATCTATCTGC	21		
**Exon5**	BCKDHB_EX5_F	GGAGGGAAAGACTTATTGTGCT	22	63.4	548
	BCKDHB_EX5_R	AACTGGGCATTGGATAGCATA	21		
**Exon6**	BCKDHB_EX6_F	CAGAACATGGTAGACATCTGA	21	59.5	553
	BCKDHB_EX6_R	AGATTTCCTCTTTGTTTCCACA	22		
**Exon7**	BCKDHB_EX7_F	CTTTGCTACAGTGAGCTTCTT	21	59.6	471
	BCKDHB_EX7_R	TAACTATGTGTGGTGATGGATC	22		
**Exon8**	BCKDHB_EX8_F	GCAGATCAGTTCCTGAGACT	20	59.6	477
	BCKDHB_EX8_R	GCATAAAGGACCCCATTTTGTA	22		
**Exon9**	BCKDHB_EX9_F	CGAAAGCGAGTTGTAACTTATTGG	24	62.5	306
	BCKDHB_EX9_R	CTTCTGGAATTGGCATGTGG	20		
**Exon10**	BCKDHB_EX10_F	GAACATGCTGTTACCTGCTT	20	57.2	390
	BCKDHB_EX10_R	CTGATGATTGCTGTGTCTTGG	21		
**3’UTR**	BCKDHB_EX11_F	AAAGAGCCAAGGTAGTGATG	20	61	694
	BCKDHB_EX11_R	CATCCTGGTCATAAAGAACTGA	22		

Supplementary Table3: PCR primers for genomic amplification of the human *DBT* exons (5’ → 3’).

Primer location	Primer Name	Primer sequence	Number of base	Annealing temperture	Amplicon size (bp)
**Exon 1**	DBT_EX1_F	CTTCCCTCCCTATTGGTCG	19	62.3	316
	DBT _EX1_R	CTCCGTTCTCTGCCCTTTATT	21		
**Exon2**	DBT _EX2_F	TCAGAAGGAATTTTGGGTAAGG	22	61	564
	DBT _EX2_R	CAATCTGGGCAACTGAGTGA	20		
**Exon3**	DBT _EX3_F	TGGAAGAATTTCTGCCTCTGCC	22	65	389
	DBT _EX3_R	TGTATGACAAAGTCCTTCACCA	23		
**Exon4**	DBT _EX4_F	CCATCTGAAAGTAAATGCTGG	21	58.3	549
	DBT _EX4_R	CCTTTTGCTATTGCCTTTTC	20		
**Exon5**	DBT _EX5_F	CCCACTCTACCCATACCATTAG	23	59.1	606
	DBT _EX5_R	AGCACCTGACATAAGACCTGG	21		
**Exon6**	DBT _EX6_F	CTGATGGTTACCACATGC	18	53.2	519
	DBT _EX6_R	TCAGTAAGACTTGAAAACACC	21		
**Exon7**	DBT _EX7_F	AGACATTAGAGAACCTTCCAT	21	58.4	306
	DBT_EX7_R2	GCTAAGGCAAGAGAATTGTTTA	22		433
**Exon8**	DBT _EX8_F	GGAACTTTGGCTGGTCTGTATC	22	63.9	660
	DBT _EX8_R	GCTGCTTCTTTTTGAGAGGGT	21		
**Exon9,10**	DBT _EX9-10_F	ATGGCAGTGAAGGTTGATCC	20	64.2	542
	DBT _EX9-10_R	TGTGTTTAGTCCCTGAATTTGCT	23		
**Exon11**	DBT _EX11_F	GGTTTGCCTGATCTTACACCA	21	62.5	490
	DBT _EX11_R	TGACCTATAAAATGTGACAGCC	23		

Supplementary Figure 1

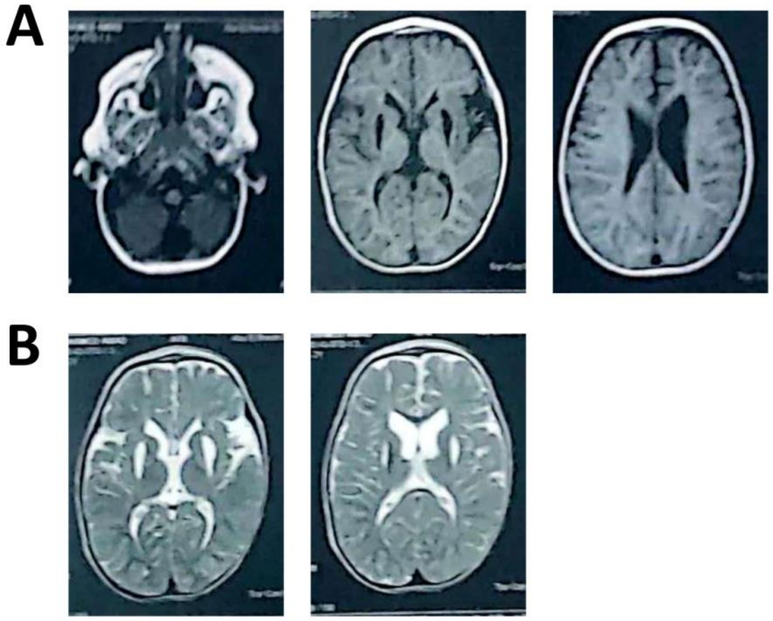

Supplementary Figure 1: Brain MRI of the proband of family VI (proband VI/7). A) Axial T1-Flair shows bilateral dilatation of subarachnoid spaces including sylvian fissures, and an insult involving bilateral basal ganglia particularly the putamen, present as marked atrophy and seen replaced by cystic encephalomalacia. The lateral ventricle show generalized mild dilatation as a result of the atrophic changes. There is also cerebellar vermian hypoplasia. **B)** Axial, T2, shows the bilateral symmetrical increased signals in the basal ganglia particularly the putamen.
